# Ambient temperature-related sex ratio at birth in historical urban populations: the example of the city of Poznań, 1848–1900

**DOI:** 10.1038/s41598-024-64799-7

**Published:** 2024-06-18

**Authors:** Grażyna Liczbińska, Szymon Antosik, Marek Brabec, Arkadiusz M. Tomczyk

**Affiliations:** 1 Institute of Human Biology and Evolution, Faculty of Biology, Adam Mickiewicz University, Poznań, Poland; 2 Doctoral School of Humanities, Adam Mickiewicz University, Poznań, Poland; 3 Department of Statistical Modelling, Institute of Computer Science, The Czech Academy of Sciences, Prague, Czech Republic; 4 Department of Biostatistics, National Institute of Public Health, Prague, Czech Republic; 5 Department of Meteorology and Climatology, Faculty of Geographic and Geological Sciences, Adam Mickiewicz University, Poznań, Poland

**Keywords:** Secondary sex ratio, Conception, Pregnancy, Temperature stress, Climate, Nineteenth century, Developmental biology, Environmental health

## Abstract

This study examines whether exposure to ambient temperature in nineteenth-century urban space affected the ratio of boys to girls at birth. Furthermore, we investigate the details of temperature effects timing upon sex ratio at birth. The research included 66,009 individual births, aggregated in subsequent months of births for the years 1847–1900, i.e. 33,922 boys and 32,087 girls. The statistical modelling of the probability of a girl being born is based on logistic GAM with penalized splines and automatically selected complexity. Our research emphasizes the significant effect of temperature in the year of conception: the higher the temperature was, the smaller probability of a girl being born was observed. There were also several significant temperature lags before conception and during pregnancy. Our findings indicate that in the past, ambient temperature, similar to psychological stress, hunger, malnutrition, and social and economic factors, influenced the viability of a foetus. Research on the effects of climate on the sex ratio in historical populations may allow for a better understanding of the relationship between environmental factors and reproduction, especially concerning historical populations since due to some cultural limitations, they were more prone to stronger environmental stressors than currently.

## Introduction

Heat stress, which is caused by a combination of ambient temperature, humidity, solar radiation, and wind speed, can cause adverse physiological responses if persists for long periods^1,2^. Numerous studies of contemporary populations indicate the influence of outdoor temperature on perinatal and pregnancy outcomes. Particularly in recent years, studies have suggested the link between extremely high ambient temperature during pregnancy and lower birth weight, and such complications as high probability of premature birth^3–7^, high prevalence of hypertension, eclampsia, and cataract in infants^8^, perinatal brain injury^9^, and the increased risk of stillbirths, miscarriages, and congenital abnormalities^10^. This problem applies to economically developing countries^2,11^ as well as to economically developed regions^8,12^. A significant increase in infant mortality at extremely high ambient temperatures was observed in Great Britain^13^, South Korea^14^, Spain^15^, and Australia^16^. In California, heat waves led to an increase in the likelihood of miscarriages, mainly of male foetuses, in the early twenty-first century. Additionally, young mothers and those with a lower socioeconomic status were more prone to a miscarriage, which the authors explain by frequent exposure to heat due to working outdoors^4^. Seasonal fluctuations in birth weight have also been demonstrated in the literature. Newborns from Aberdeen, Scotland, born in the years 1950–1956 in the winter months had the lowest birth weight, while those born in the autumn months—had the highest birth weight. Higher ambient outdoor temperature in the first trimester of pregnancy and/or lower ambient outdoor temperature in the third trimester of gestation were associated with reduced offspring birthweight^17^. Infants born in Ireland in 1971–1986, during late spring and summer were lighter than those born in winter, which according to the authors, might have resulted from the exposure to low winter temperatures during mid-gestation^18^.

Researchers have also highlighted the role of ambient temperature as the factor influencing the sex ratio at birth, i.e. the ratio of boys to girls at birth (so-called: secondary sex ratio; SSR). According to biological law, 105–107 boys come into the world per 100 girls born alive^19^. It was found that the sex ratio at birth is shaped by ambient temperature fluctuations^20^. The proportion of boys to girls at birth rises with warmer ambient temperatures and declines with colder ones^20–22^. Spatial analyses of selected world populations from late twentieth century showed that in Europe, more males than females were born in southern latitudes than in northern ones, while in North America it was the other way around^23^. Research by Helle and a team^24^ on three Sami populations, in Finland, from the eighteenth to nineteenth centuries suggested again that a warm climate may bring more sons than daughters. In turn, higher temperatures in the previous year increased female births^24^. Catalano and colleagues^20^, using time series, showed that pregnancies occurring at low ambient temperatures predict lower male-to-female sex ratios in birth cohorts consisting of Danes, Finns, Norwegians, and Swedes born between 1878 and 1914.

Temperatures, both high and low, are considered environmental stressors. They affect the human body both directly and indirectly, through droughts, heavy rainfall, and weather anomalies that limit harvests and therefore, affect the local and national economy, standard of living, etc. The present study examines whether exposure to ambient temperatures in nineteenth-century urban space affected the secondary sex ratio. We predict that ecological stress caused by ambient temperatures during pregnancy significantly reduces the ratio of male-to-female live births, irrespective of seasonal fluctuations of the secondary sex ratio (SSR). Furthermore, we investigate the details of temperature effects timing upon sex ratio at birth. Research on the effects of climate on the sex ratio in historical populations may allow for a better understanding of the relationship between environmental factors and reproduction, especially in relation to historical populations since they were more prone to environmental stressors. Changes in ambient temperature have influenced what foetus would survive gestation, and this affected the characteristics of human populations in the past. The changes in outdoor temperature over time could have had important implications for public health, labour market, insurance policy, and other areas. To our knowledge, this is the first study to examine the effect of temperature on the boy-to-girl live birth ratio in historic urban space. In addition, we analyse in detail the time-lag structure of the effect statistically.

## Results

In Model 0, which estimated the effect of birth year and birth month on the probability of giving birth to a girl, the seasonal effects (annual and month effects) were not significant. Next, we added the mean temperature in the year of conception to the components in Model (M0), obtaining (MT0). The temperature in the year of conception was statistically significant (Table 1).

**Table 1:** Parametric and non-parametric smooth effects for birth year, birth month, and mean annual temperature in the year of conception on the probability of giving birth to a girl.

Summary of parametric effects			
Effect name	Estimate	Standard error	p-value
Intercept	−0.058	0.008	0.000***

Smooth effects			
Effect name	Equivalent degrees of freedom (effect complexity)	p-value	
Birth year	1.573	0.553	
Birth month	0.000	0.923	
Temperature in the year of conception	1.087	0.004**	

***p < 0.001; **p < 0.01.

**Figure 1:**
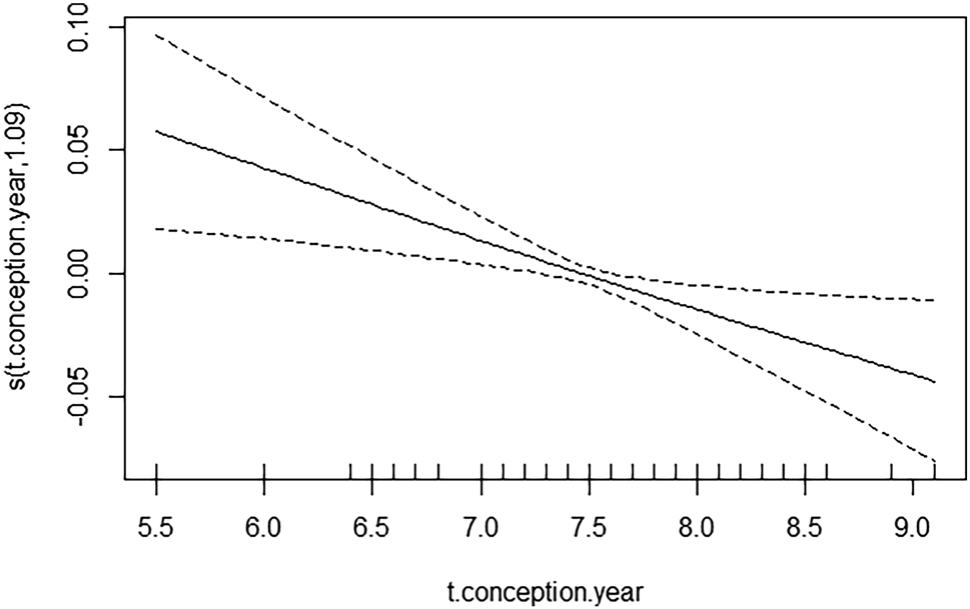
The effect of an average temperature in the year of conception on the probability of giving birth to a daughter. The figure based on data collected for the project supported by the National Science Centre, Poland. Grant: *Long-term social costs of adverse conditions occurring in early life in the 19th-century populations on the example of the city of Poznań,* No. UMO-2020/39/O/HS3/00524.

**Figure 2:**
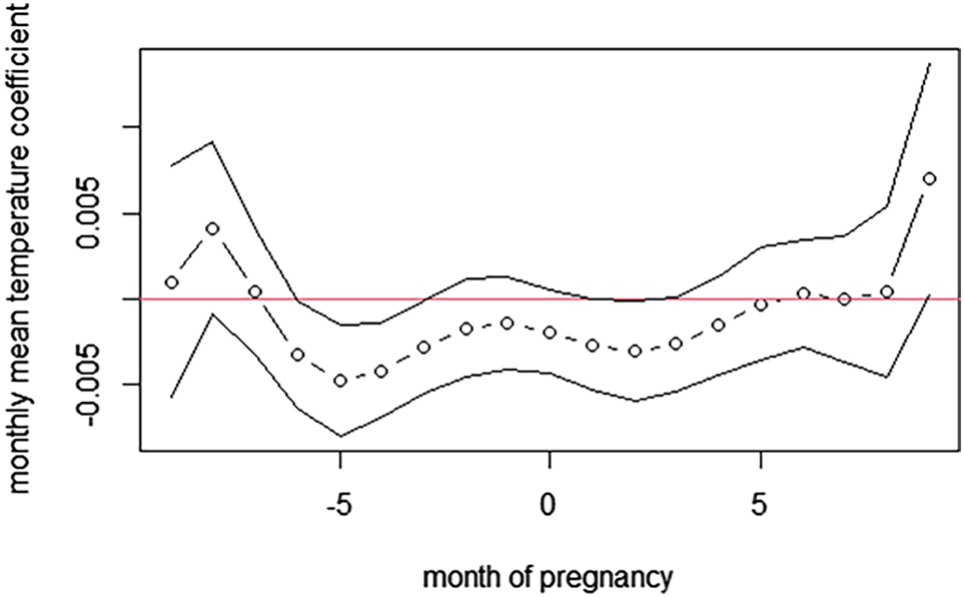
The effect of an average temperature in the months before and after conception on the probability of giving birth to a daughter. The figure based on the data collected for the project supported by the National Science Centre, Poland. Grant: *Long-term social costs of adverse conditions occurring in early life in the 19th-century populations on the example of the city of Poznań,* No. UMO-2020/39/O/HS3/00524.

The effect was negative and nearly linear (Fig. 1), which means that the higher the temperature, the smaller probability of a girl being born was observed. The more elaborate model (MTA) described the more detailed Almon lag logistic GAM model with monthly temperature averages for months, such as −9, −8, −7, −6, −5, −4, −3, −2, −1, 0, 1, 2, 3, 4, 5, 6, 7, 8, 9 with respect to the month of conception (before and after conception) (Table 2; Fig. 2). There were several significant temperature lags: −6, −5, −4, −3 (before conception), and 2 (during pregnancy).

**Table 2:** The effects of mean monthly temperatures lagged −9…to 0 and 0…to 9 concerning the month of conception on the probability of giving birth to a girl.

Lag	Coefficient	Standard error	L	U
−9	0.0009	0.0034	−0.0058	0.0078
−8	0.0041	0.0025	−0.0008	0.0091
−7	0.0004	0.00189	−0.0032	0.0040
−6	−0.0033*	0.0016	−0.00644	−0.0002
−5	−0.0047*	0.0016	−0.0080	−0.0015
−4	−0.0042*	0.0014	0.0014	−0.0017
−3	−0.0028*	0.0014	−0.0055	−0.0001
−2	−0.0017	0.0015	−0.0046	0.0011
−1	−0.0014	0.0014	−0.0041	0.0012
0	−0.0019	0.0012	−0.0044	0.0005
1	−0.0027	0.0014	−0.0054	0.0000
2	−0.0031*	0.0015	−0.0059	−0.0002
3	−0.0027	0.0014	−0.0054	0.0000
4	−0.0016	0.0014	−0.0044	0.0013
5	−0.0003	0.0015	−0.0036	−0.0029
6	0.0003	0.0016	−0.0029	0.0034
7	−0.0000	0.0018	−0.0036	0.0036
8	0.0004	0.0025	−0.0046	0.0054
9	0.0069	0.0034	0.0002	0.0137

L and U correspond to the lower and upper limits of (pointwise) 95% confidence intervals.

*Statistically significant.

## Discussion

### The impact of ambient temperature on the physiology of reproduction

Temperature-dependent sex determination is well documented in vertebrates such as reptiles, fish, and some amphibians. It is related to the developmental growth rate^22,25^. A similar relationship is also observed in humans. This is supported by studies of modern populations, suggesting that in warmer months/higher temperatures more sons are produced than daughters^20–23^. In higher temperatures, male foetuses develop faster than female ones^22^. Therefore, males outnumber females in hot climates^26^. It is also suggested that the genome contains genes that determine the child’s sex, the so-called “sex-determining region on the Y chromosome”, SRY. These genes trigger the expression of several genes involved in the development of the male gonad^27^. It is suggested that these genes may be activated by temperature^22^. In stressful conditions, stress hormones produced by mother’s body disrupt mechanisms related to the functioning of SRY protein that determines the embryogenesis of male foetuses^28^. Moreover, research conducted on the populations of economically developing countries, proves that the sex ratio is influenced by the temperature at the time of conception and in very early pregnancy: the higher the temperatures at the time of conception, the higher the rate of masculinization of newborns^29^. Hajdu and Hajdu^30^ reached similar conclusions when examining births in Hungary in the years 1980–2015. Additionally, researchers report that exposure to hot temperatures changes the timing of some conceptions, i.e., some pregnancies might experience a shift in conception^30^.

Our study verified whether ambient temperatures could have affected the proportion of boys to girls at birth. The results show that introducing the average temperature in the year of conception into the model has a significant impact on the proportion of boys to girls at birth: the higher the temperature, the lower the probability of giving birth to a girl, while the probability of giving birth to a boy increased, and the other way around. The situation is similar when average monthly temperatures for the months before conception and during foetal development (during pregnancy) are introduced into the model. Although higher air temperatures may lead to the increased likelihood of having a boy, such extremely high temperatures may have the opposite effect and shift the sex ratio towards females (lower sex ratio values). Research shows that ambient temperature may influence primary sex determination through variable fertilization success with sperm with X or Y chromosomes^22^. Temperature influences the course of spermatogenesis and oogenesis^31,32^. In mammals, high ambient temperature affects the concentration of steroids in ovarian follicles^33^. These endocrine changes reduce the activity of follicles and change the mechanism of ovulation, which leads to a decrease in the quality of oocytes and embryos. The uterine environment also changes, which reduces the probability of embryo implantation and increases the risk of embryo loss^32^. The function of the uterine endometrium is also impaired and its secretory activity changes, which may lead to termination of pregnancy^33^. Hyperthermia during pregnancy can cause embryo’s death, miscarriage, growth retardation, and developmental defects^2,34^. Extremely high temperatures affect placental blood flow and increase the risk of hypertensive crises and stillbirth by increasing vasoactive substance production, blood viscosity, and endothelial cell activity^35^. Environmental stresses may also impair sperm motility, potentially promoting transition to female sex at birth^36–38^. Studies conducted on experimental animals suggest that males exposed to high temperatures are less fertile^39,40^, which is due to poorer semen quality^39,40^. The effect of heat exposure on semen quality is transient, producing the strongest effects not immediately after heat stress, but several weeks later^41–43^.

In turn, exposure to low temperature increases plasma fibrinogen levels, increases blood viscosity, and may cause placental vasoconstriction, reducing uteroplacental blood flow and inhibiting foetal growth^18^. Several other processes may occur during winter that may have detrimental effects on foetal development, such as increased exposure to infections, decreased physical activity, increased exposure to pollutants, and increased pregnancy-induced hypertension^18^.

The high mortality of more sensitive male foetuses during temperature stress may have contributed to the reduction in the ratio of live-born boys to girls in conditions of extremely high/low temperatures^20,26^. According to estimates, environmental stresses can lead to the loss of at least 70% of conceptions (they are miscarried before delivery)^44–46^. In the light of the evolutionary theory, fewer males should be born during stressful periods because the weaker males would not survive and be unable to reproduce where the females could reproduce^47^. It is known that the male foetus is more susceptible to environmental insults, and indeed the male foetus is generally more fragile and prone to be stillborn^26^.

### Ambient temperature values in historical Poznań and their link to sex ratio at birth

In Poland, the temperature trend has been systematically increasing since the middle of the nineteenth century. The last two decades of the twentieth century and the first decade of the twenty-first century are the warmest in the 230-year history of meteorological observations in the country. The extreme weather phenomena, which earlier occurred sporadically, have recently become more frequent and intense^48^. It is worth remembering that Poles have slightly lower chances in the clash with heat waves in comparison with the inhabitants of southern Europe. They usually have slightly brighter skin, are more susceptible to burns, and do not have air-conditioning systems in homes^49^. Hence, a rapid response of an organism to extremely high temperatures often leads to physiological disorders and/or also to a direct impact on health status in the form of asthma, allergies, and death-related complications^50^.

**Figure 3:**
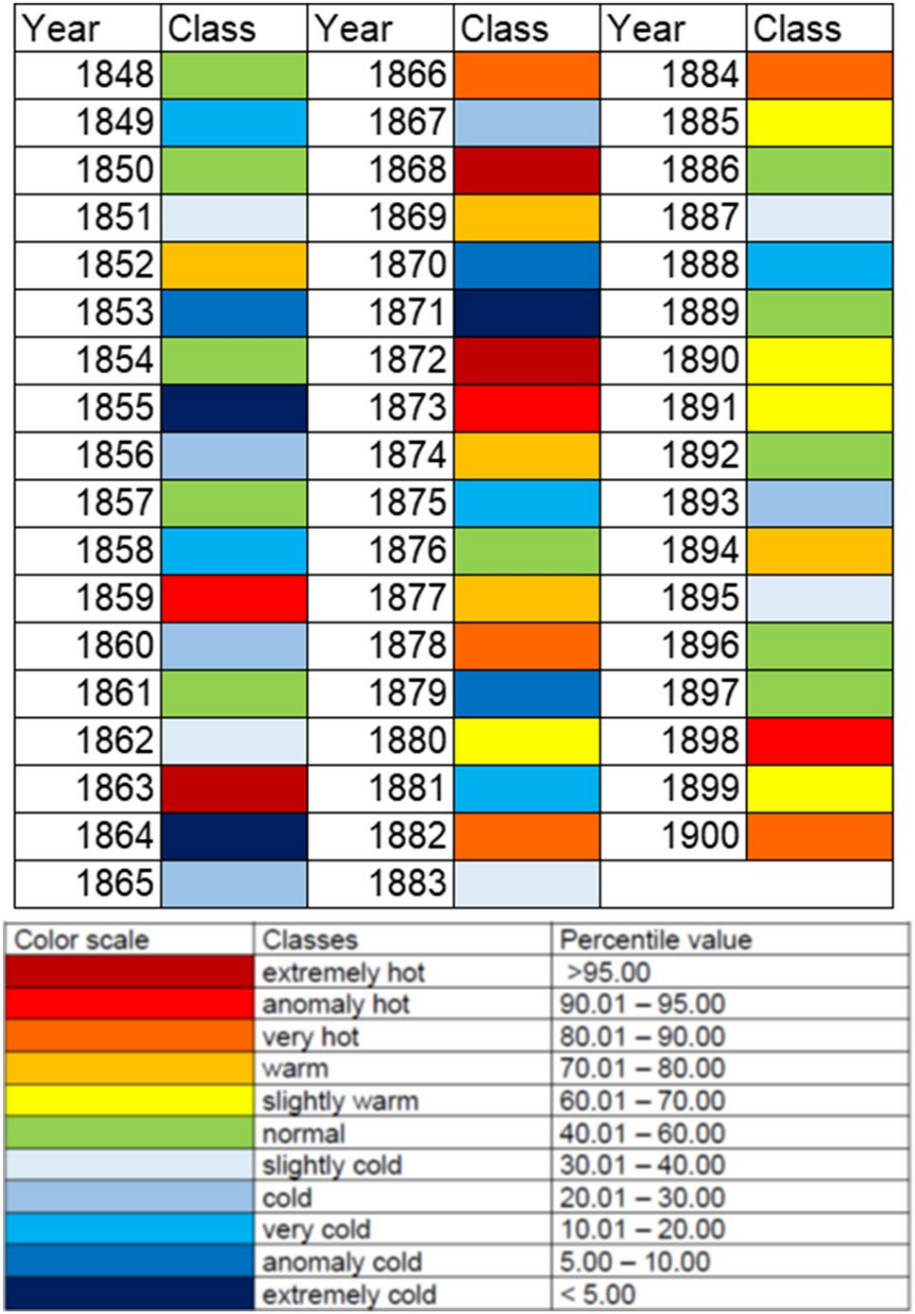
The percentile classification of the annual mean temperature. Thresholds of 5, 10, 20, 30, 40, 60, 70, 80, 90, and 95 percentiles denote 11 classes (described in the legend). Classes were calculated for the reference period of 1848–1900. In the table, the criteria for thermal quantile classification of month and years.

Let us recall once again that in our research, introducing the average temperature in the year of conception into the model significantly affects the probability of giving birth to a boy. The situation is similar when average monthly temperatures for the months before conception and during foetal development (during pregnancy) are introduced into the model. It suggests a positive impact of warm months on conception and foetal development and the increase in the ratio of live-born boys to girls, as discussed in the previous chapter. Climatological studies of nineteenth-century Poznań (see: “Results”; Fig. 3) indicate that in the years 1848–1900 the average annual temperature categorized as “normal” occurred 11 times, the average annual temperatures categorized as “light warm” was recorded in the following years: 1880, 1885, 1890, 1891 and 1899 (Fig. 3), and the average annual temperatures categorized as “warm” occurred in the years: 1852, 1869, 1874, 1877 and 1894 (Fig. 3). These temperatures may have favoured male conceptions through mechanisms described in the previous chapter of this work. McLaren and Wilde^29^ drew attention to the relationship between temperatures in the months around conception and the child’s sex. At the same time, researchers emphasized that the results of these studies apply to economically developing countries. In developed countries where it is possible to regulate indoor temperature, for example, by air conditioning, researchers have found no effect of ambient temperature at conception and during pregnancy on the sex ratio^29^. Poznań in the nineteenth century, in terms of infrastructure development, corresponded to some extent, to some modern economically developing countries.

In the period under study, Poznań experienced a rapid demographic development, resulting in the increase in population from 44,000 inhabitants in the mid-nineteenth century to 117,000 at the beginning of the twentieth century. At the end of the nineteenth century, Poznań was the sixth most densely populated city in the Prussian Empire^51^. On the outskirts of the city, tenement houses were built for poorer tenants, who were mainly immigrants coming to the city for work^52^. The small, stuffy, and humid apartment was occupied by several to a dozen people^52^. In 1871 in Poznań, one residential building was populated on average by almost 40 people, and the average household had over five people^53^. Therefore, it is not surprising that the living conditions in nineteenth-century Poznań are described as terrible; Poznań residents struggled with the problem of high rents, overcrowded, cramped, damp, unheated, and poorly lit apartments^51,52^. At the beginning of the twentieth century, the fee for renting one room consumed from 40 to 56% of the income of the lowest earners^54^. There was little left to cover the costs of food, fuel, light, or clothing. The lowest rent was equal to the two-week earnings of an unskilled worker, and the highest rent exceeded their salary. Usually, one room was rented, due to prices—in the attic or basement. In 1900, over 10,000 people lived in one-room basements and attics, i.e., 1/10 of the city inhabitants^54^. It is worth adding that in the same year, almost 45% of Poznań inhabitants lived in one-room apartments: overcrowded, damp, and unheated^54^. Everyday life in such conditions was particularly burdensome in autumn, winter, and early spring when residents were bothered by cold and damp rooms, especially when the temperature was low or extremely low. Summer months were also difficult with high and extremely high-temperature values, when the rooms became stuffy, and the lack of hygiene and sewage system resulted in an epidemiological threat.

Working conditions also left much to be desired. A 12-h workday was common, sometimes extended to 16 h, without additional remuneration^54^. The workers stayed on the factory premises for an additional hour to over 2 h longer^54^. A significant percentage of the professionally active population in Poznań in the second half of the nineteenth century were low-skilled workers whose working conditions were often physically and mentally exhausting^55^. Work was often done at home as cottage industry, which, given the poor sanitary standard of Poznań apartments, did not have a positive effect on health. Workplaces often did not meet sanitary standards^55^. Many of the labourers worked in cramped and low rooms, which were damp and lacked ventilation. The lack of adequate airflow was especially bothersome for workers in craft workshops and factories, where there was also the problem of dust, and the factory halls were crowded with equipment, waste, and people^55^. Labourers working in mineral, food, and metallurgy industries especially had to struggle with high temperatures. This was particularly hard during hot weather, when employers, against legal regulations, did not provide adequate access to clean drinking water^55^. Women could enjoy some improvement in working conditions, as Prussian legislation introduced solutions in the last decade of the nineteenth century that were aimed at improving their working conditions, mainly by shortening the working day. Mandatory breaks were also introduced, and women’s work was prohibited, among others, during night shifts and on Sundays and holidays^55^.

Our findings do not directly contribute to estimates of the effects of ambient temperature values on the proportion of boys to girls at birth. However, they indicate that in the past, ambient temperature, as well as psychological stress, hunger, malnutrition, and social and economic factors, influenced the viability of the foetus. The results, however, suggest a heretofore unrecognized impact of temperature on reproduction (who survives gestation) in past societies. As research shows that temperatures have been rising in Poland since the nineteenth century^49^, our results begin to track the impact of climate change on the sex ratio at birth. Our findings are based on data from Poznań, a city located in Central Europe. Therefore, the results can be generalized to other countries from this geographical and climatic zone from a similar period because the climatic conditions in many countries of Central and Eastern Europe are very similar. Moreover, it will be interesting to use the same deeper temperature-lag-effect analysis (based on the Almon lags) in different parts of the world to compare the profile of the temperature-effect trace. Our results show that in past and present-day populations, ambient temperature had implications for public health.

## Material and methods

### Temperature observations in Poznań in the nineteenth century

The first temperature measurements began in Poznań in 1848, in the meteorological station at 1 Pocztowa Street, moved then to Pocztowa Street, and then to 1 Grobla Street. In the latter place, the thermometer was set at 2.5 m above ground level (AGL)^56^. In August 1885, the location of the station was changed from 1 Zielona Str. to 2 Zielona Str., where temperatures were measured with a thermometer set at 6.2 m AGL^56^. In September 1892 the station moved to 3 Długa Street and the thermometers were placed at 8.6 m AGL. Here the station operated until December 1911^56^. Until 1892, temperature measurements were taken with a thermometer without a cover and placed outside the window. Since September 1892, zinc sheet sheaths were used on thermometers to protect the measurements against atmospheric factors such as wind, sun, and humidity, which distorted the results^56^. Until 1884, measurements were taken three times a day: at 6 am, 2 pm, and 10 pm. Between December 1884 and May 1919, the hours of observation were changed for 7 am, 2 pm, and 9 pm. Meteorological observations in Poznań were carried out by professors at the Municipal Real School: first by Spiller, and since April 1862—by Magener. In 1889, meteorological measurements were taken by the Physical Institute of the Royal Academy^56^. Since the 1890s, a meteorological station operated also in the village of Jeżyce where the thermometers were set at 9.5 m AGL. A pharmacist Wildt was the manager of the station. Here the meteorological observations were taken three times a day: at 7 am, 2 pm, and 9 pm^56^. Initially, temperature was measured using the Reamur scale. The Celsius scale has been used since 1879. The daily temperature was the arithmetic mean of three daily observations^56^.

### The changes in outdoor temperature in Poznań in the second half of the nineteenth century

**Figure 4:**
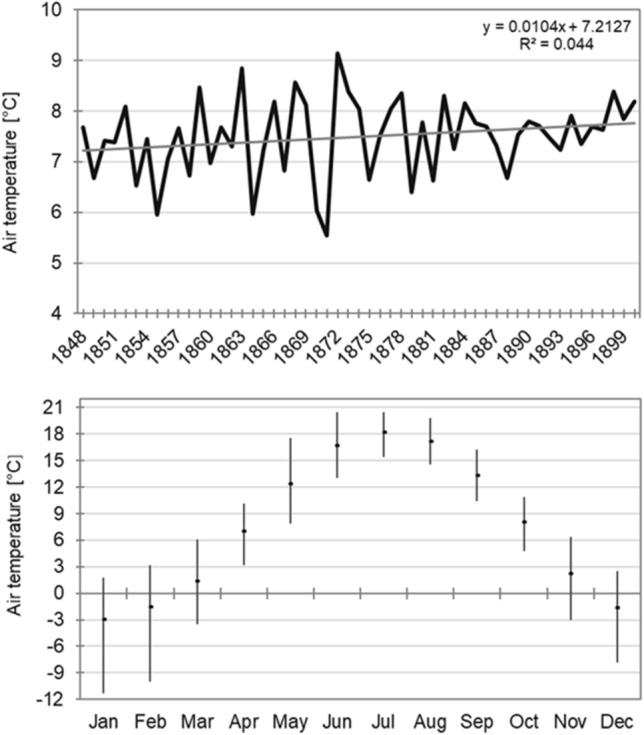
Multiannual course of the average annual air temperature (top) and monthly course of the air temperature (dots), verticals indicate a range of the average monthly air temperature changes (bottom).

Thermal conditions in Poznań in 1848–1900 were determined based on mean monthly air temperature values obtained from the work by Kolendowicz and a team^57^. The data made it possible to calculate a mean annual air temperature, as well as monthly means and the annual mean for the entire multiannual period. Then, the direction of changes and their statistical significance were analysed using a t-student test (significance level 0.05). In the next step, the classification of thermal conditions in particular months and years was conducted. The adopted classification is based on percentiles calculated for mean monthly (each separately) and annual values, with consideration of the following thresholds: 5, 10, 20, 30, 40, 60, 70, 80, 90, and 95 percentiles. The resulting 11 classes were calculated for the period 1848–1900 and adopted for the development of a heat map (Fig. 3). In the years 1848–1900 in Poznań, the mean annual air temperature was 7.5 °C. In the studied period, it varied from 5.5 °C in 1871 to 9.1 °C in 1872 (Fig. 4). These data suggest that in the analysed years, deviations from the multiannual mean reached −2.0 °C and 1.6 °C, respectively. In the analysed period, a high variability of mean air temperature was recorded. A relatively high standard deviation (0.8 °C) shows a broad distribution of mean values for particular years around the multiannual mean. A year with a high mean air temperature can occur right after a year with a low value, and vice versa. High variations in thermal conditions were observed in the 1860s and 1870s when extremely cold years were recorded (1855, 1864, 1871) following extremely warm years (1863, 1868, 1872) and vice versa (Fig. 3). The conducted research showed an increase in the mean annual air temperature at a level of 0.1 °C/10 years, although the changes were not statistically significant. In the analysed multiannual period, the mean monthly air temperature varied from −3.0 °C in January to 18.1 °C in July. Apart from January, mean monthly values below 0 °C were also recorded in February and December (−1.6 °C) (Fig. 4). The greatest range of variation of mean monthly air temperature was recorded in the winter months, and the lowest in the summer months. Standard deviation values ranged from 1.2 °C in August to 3.0 °C in January and February. Like in the case of the mean annual air temperature, high year-to-year variations were also observed from month to month in a year. The lowest mean monthly air temperature was −11.3 °C and occurred in January 1848. Air temperature below −10.0 °C was still recorded in January 1893 (−10.5 °C). The highest mean monthly air temperature was 20.5 °C, recorded in June 1889 and July 1865. Moreover, the value of 20.0 °C was exceeded in July 1874 and 1900. In the analysed multiannual period, except for June and October, an increase in mean monthly air temperature was observed, although the changes were not statistically significant. The most intensive increase was recorded in November (0.28 °C/10 years) and January and March (0.23 °C/10 years).

### Ethics statement

The work uses nineteenth-century archival data (publicly available) deposited in the form of parish birth books in the State Archives in Poznań, also available online at: https://poznan.ap.gov.pl/zasob-2/zasob-online/szukaj-w-archiwach/swa/.

The processing of nineteenth-century data derived from archives is not covered by data protection provisions, so it does not require consent from ethical committees.

### Dataset

We used individual information on live births gathered from the parish birth registers of seven Poznań parishes (St. Mary Magdalene, St. Margaret, St. Martin, St. Adalbert, St. John, Holy Cross, and St. Paul). The data included individuals born to mothers residing in Poznań. All births took place in Poznań. We have assumed that maternal place of residence was her place of pregnancy. In total, the material included 66,009 numbers of births with known months of birth, which were aggregated in subsequent months of births for the years 1847–1900. The final dataset consisted of 33,922 boys and 32,087 girls. The number of births in each month of a given year was assigned the average monthly temperature for that month. The next step was to estimate the month of conception. The aggregate data do not include the exact date of birth. Thus, the date of conception (the beginning of intrauterine development) was estimated based on information regarding the month of birth, assuming conventionally the 15th day of each month as the date of birth and 280 days (40 weeks) as the average length of human gestation^58,59^:

$$time \,of \,conception=15th \,day \,of \,a \,given \,month-280 \,days \,of \,pregnancy.$$

Each month of conception was also assigned the average temperature in that month (mon[0]) and the average temperatures in the months subsequent to (mon[1]to mon[9]) and prior to (mon[−9] to mon[−1]) pregnancy. Homogenized temperature data in the years 1848–1900 were taken from the work of Kolendowicz and a team^57^, published in *Theoretical and Applied Climatology* in 2019, attached by the authors in an open file.

## Methods

The statistical modelling of the probability of a girl being born is based on logistic GAM (Generalized Additive Model^60,61^) with penalized splines and automatic complexity selection^62^. First, we fit a model (M0) that estimates the classical trend and seasonal decomposition of SSR time series, where both components are fully nonparametric (unlike in many traditional analyses). Then we investigate temperature effects on the probability of a girl being born (PGB) in several specialized models. The simplest (MT0) adds mean annual temperature (in the calendar year of birth) to the previous trend and seasonal components to describe possible additional colder/warmer weather effects on a long-term (annual) scale only. Further, we use more detailed analyses focusing on temperature effects closer to the birth date month (before, at, and after). The approach is based on the Almon distributed lag^63^ sub-model instead of the annual temperature average. This sub-model allows us to address the impact of differently distant pasts upon the PGB. Namely, we estimate coefficients (loosely interpretable as “weights”) on monthly-averaged temperature for several months before the birth date simultaneously. Estimating these coefficients is notoriously problematic or even impossible to conduct directly (due to their heavy inter-correlations) and a regularization of this ill-posed problem is necessary. To this end, we use a classical approach developed in econometrics and now used in other fields^64,65^. Specifically, we use the model (MTA) with the Almon polynomial sub-model for addressing simultaneously the monthly temperature average effects with the lags of −9, −8, −7, −6, −5, −4, −3, −2, −1, 0, 1, 2, 3, 4, 5, 6, 7, 8, 9 with respect to the conception. All lags are before the birth date. Negative lags correspond to the months before conception, while positive ones to the months after conception and before birth.

The annual trend was modelled nonparametrically (as a complexity penalized spline of disease-group specific shape). One of the main advantages of this approach is that it can capture complicated nonlinear shapes of trends, including periodic behaviour if the data support them. Computations were done in the R environment^66^.

All methods were carried out in accordance with relevant guidelines and regulations.

### Methodological limitations

The methodological tools used in this work have certain limitations. The first is related to the use of aggregated data which contains information about the number of live births in particular months of the year only. This involved estimating the moment of conception, and based on which the months of gestation with exposure to ambient temperatures were established. Thus, the distinguished months of pre-conceptions/conceptions/pregnancies could have been consistently affected by an error resulting from the previous estimates. Moreover, foetuses in gestation in one year could have been born in the next. The aggregated data did not enable a deeper insight into the studied phenomenon, which would have been possible if the information on individual births had been used. Individual data could have allowed the partitioning of gestation into trimesters and to capture the developmental stage which was the most sensitive to ambient temperatures. Secondly, the aggregated data on live births by sex in particular months of the year do not contain information on the duration of pregnancy, including pre-term and prolonged pregnancies. We assume that all pregnancies are terminated on time. In contemporary Poland, 92.4% of pregnancies end on time^67^, while only 7.6% of them are pre-term and post-term (the 7.6% of pre- and post-term pregnancies did not burden the results with an error).

Thirdly, lack of information on foetal deaths in the uterus, miscarriages/abortions, and stillbirths did not make it possible to capture the factors driving the decline/increase in SSR in some months or years in the period under study. The fourth limitation stems from the lack of data on daily temperatures. In this paper, we used average monthly and yearly temperatures.

Moreover, the sex ratio at birth may have been affected by biological factors. One of them is gestational age, GA. Weng and a team show that the sex ratio decreased proportionally with increasing GA^68^. Another factor influencing the sex ratio is birth order^69^: nulliparous increases the chances of delivering a boy^70^. Birth may be related to maternal age^70^. The mothers' socioeconomic factors, such as maternal marital status and type of job could also have been confounding factors. The exposure of unmarried pregnant women to stress resulting from financial problems, poor living conditions, unemployment, lack of psychosocial support social instability, and physical workload may have resulted in the elimination of weaker male fetuses more often than female ones^71,72^.

## Data Availability

Collection of the data on mortality was supported by the National Science Centre, Poland, under Grant *Long-term social costs of adverse conditions occurring in early life in the 19th-century populations on the example of the city of Poznań,* No. UMO-2020/39/O/HS3/00524. The dataset used in this study is available at https://data.mendeley.com/datasets/n4jdgfxmwv/1: Liczbińska, Grażyna (2024), “Ambient temperatures in 19th-century Poznan and sex ratio at birth”, Mendeley Data, V1, 10.17632/n4jdgfxmwv.1. Data on homogenized temperatures in 1848–1900 were taken from the file attached as an open file to the work: Kolendowicz et al.^57^.

## References

[1] Bonell A, Hirst J, Vicedo-Cabrera AM, Haines A, Prentice AM, Maxwell NS (2020). A protocol for an observational cohort study of heat strain and its effect on fetal wellbeing in pregnant farmers in The Gambia. Wellcome Open Res..

[2] Rekha S, Nalini SJ, Bhuvana S, Kanmani S, Vidhya VA (2023). Comprehensive review on hot ambient temperature and its impacts on adverse pregnancy outcomes. J. Mother Child.

[3] Flouris AD, Spiropoulos Y, Sakellariou GJ, Koutedakis Y (2009). Effect of seasonal programming on fetal development and longevity: Links with environmental temperature. Am. J. Hum. Biol..

[4] Basu R, Sarovar V (2016). Association between high ambient temperature and risk of stillbirth in California. Am. J. Epidemiol..

[5] Rylander C, Odland JØ, Sandanger TM (2011). Climate change and environmental impacts on maternal and newborn health with focus on Arctic populations. Glob. Health Action.

[6] Auger N, Naimi AI, Smargiassi A, Lo E, Kosatsky T (2014). Extreme heat and risk of early delivery among preterm and term pregnancies. Epidemiology.

[7] Kuehn L, McCormick S (2017). Heat exposure and maternal health in the face of climate change. Int. J. Environ. Res Public Health.

[8] Shashar S, Kloog I, Erez O, Shtein A, Yitshak-Sade M, Sarov B, Novack L (2020). Temperature and preeclampsia: Epidemiological evidence that perturbation in maternal heat homeostasis affects pregnancy outcome. PLoS One.

[9] Kasdorf E, Perlman JM (2013). Hyperthermia, inflammation, and perinatal brain injury. Pediatr. Neurol..

[10] Asamoah B, Kjellstrom T, Östergren P-O (2018). Is ambient heat exposure levels associated with miscarriage or stillbirths in hot regions? A cross-sectional study using survey data from the Ghana Maternal Health Survey 2007. Int. J. Biometeorol..

[11] Rahman J, Fakhruddin S, Rahman AF, Halim M (2016). Environmental heat stress among young working women: A pilot study. Ann Glob. Health.

[12] Vicedo-Cabrera AM, Olsson D, Forsberg B (2015). Exposure to seasonal temperatures during the last month of gestation and the risk of preterm birth in Stockholm. Int. J. Environ. Res. Public Health.

[13] Rooney C, McMichael A, Kovats R, Coleman M (1998). Excess mortality in England and Wales, and in Greater London, during the 1995 heat wave. J. Epidemiol. Commun. Health.

[14] Kysely J, Kim J (2009). Mortality during heat waves in South Korea, 1991 to 2005: How exceptional was the 1994 heat wave?. Clim. Res..

[15] Basagña X, Sartini C, Barrera-Gómez J, Dadvand P, Cunillera J, Ostro B, Sunyer J, Medina-Ramón N (2011). Heat waves and cause-specific mortality at all ages. Epidemiology.

[16] Nitschke M, Tucker GR, Hansen AL, Williams S, Zhang Y, Bi P (2011). Impact of two recent extreme heat episodes on morbidity and mortality in Adelaide, South Australia: A case series analysis. Environ. Health.

[17] Lawlor, D. A., Leon, D. A. & Smith, G. D. The association of ambient outdoor temperature throughout pregnancy and offspring birthweight: Findings from the Aberdeen Children of the 1950s cohort. *BJOG1* **12**(5), 647–657 10.1111/j.1471-0528.2004.00488.x (2005). 10.1111/j.1471-0528.2004.00488.x 15842292

[18] Murray LJ, O’Reilly DP, Betts N, Patterson CC, Davey Smith G, Evans AE (2000). Season and outdoor ambient temperature: Effects on birth weight. Obstet. Gynecol..

[19] Cavalli-Sforza LL, Bodmer WF (1971). The Genetics of Human Populations.

[20] Catalano R, Bruckner T, Smith KR (2008). Ambient temperature predicts sex ratios and male longevity. PNAS.

[21] Lerchl A (1999). Sex ratios at birth and environmental temperatures. Naturwissenschaften.

[22] McLachlan JC, Storey H (2003). Hot male: Can sex in humans be modified by temperature?. J. Theor. Biol..

[23] Grech V, Savona-Ventura C, Vassallo-Agius P (2002). Unexplained differences in sex ratios at birth in Europe and North America. BMJ.

[24] Helle S, Helama S, Jokela J (2008). Temperature-related birth sex ratio bias in historical Sami: Warm years bring more sons. Biol. Lett..

[25] Murray, J. D., Deeming, D. C. & Ferguson, M. W. J. Size-dependent pigmentation-pattern formation in embryos of alligator mississippiensis: Time of initiation of pattern generation mechanism. *Proc. R. Soc. B. Biol. Sci.* **239**(1296), 279–293 http://www.jstor.org/stable/49461 (1990). 10.1098/rspb.1990.0017 1972793

[26] Grech V, Vassallo-Agius P, Savona-Ventura C (2000). Declining male births with increasing geographical latitude in Europe. J. Epidemiol. Commun. Health.

[27] Schmahl J, Capel B (2000). Cell proliferation is necessary for the determination of male fate in the gonad. Dev. Biol..

[28] Piprek RP, Kubiak JZ (2019). Historia badań nad determinacją płci. Kosmos.

[29] McLaren, Z. & Wide, J. *The Effect of Ambient Temperature during Pregnancy on Human Sex Ratios at Birth*. https://paa.confex.com/paa/2017/mediafile/ExtendedAbstract/Paper16645/McLaren_Wilde_Temperature_Gender_9292016.pdf. Accessed 1 March 2024 (2016).

[30] Hajdu T, Hajdu G (2022). Temperature, climate change, and human conception rates: Evidence from Hungary. J. Popul. Econ..

[31] Rutledge JJ, Monson RL, Northey DL, Leibfried-Rutledge ML (1999). Seasonality of cattle embryo production in a temperate region. Theriogenology.

[32] De Rensis F, Scaramuzzi RJ (2003). Heat stress and seasonal effects on reproduction in the dairy cow—A review. Theriogenology.

[33] Wolfenson D, Roth Z, Meidan R (2000). Impaired reproduction in heat-stressed cattle: Basic and applied aspects. Anim. Reprod. Sci..

[34] Edwards MJ, Saunders RD, Shiota K (2003). Effects of heat on embryos and foetuses. Int. J. Hyperthermia.

[35] van Zutphen AR, Lin S, Fletcher BA, Hwang S-A (2012). A population based case–control study of extreme summer temperature and birth defects. Environ. Health Perspect..

[36] Fukuda M, Fukuda K, Shimizu T, Møller H (1998). Decline in sex ratio at birth after Kobe earthquake. Hum. Reprod..

[37] Fukuda M, Fukuda K, Shimizu T, Yomu-Ra W, Shimizu S (1996). Kobe earthquake and reduced sperm motility. Hum. Reprod..

[38] Gomendio M, Malo AF, Soler AJ, Fernández-Santos MR, Esteso MC, García AJ, Roldan ER, Garde J (2006). Male fertility and sex ratio at birth in red deer. Science.

[39] Paul C, Murray AA, Spears N, Saunders PTK (2008). A single, mild, transient scrotal heat stress causes DNA damage, subfertility and impairs formation of blastocysts in mice. Reproduction.

[40] Yaeram J, Setchell BP, Maddocks S (2006). Effect of heat stress on the fertility of male mice in vivo and in vitro. Reprod. Fertil. Dev..

[41] Brito LFC, Silva AEDF, Barbosa RT, Unanian MM, Kastelic JP (2003). Effects of scrotal insulation on sperm production, semen quality, and testicular echotexture in *Bos indicus and Bos indicus* × *Bos taurus bulls*. Anim. Reprod. Sci..

[42] Garcia-Oliveros LN, de Arruda RP, Batissaco L, Guilger Gonzaga VH, Moreira Nogueira VJ, Florez-Rodriguez SA, dos Santos Almeida F, Rodrigues Alves MB, Costa Pinto SC, Nichi M, de Agostini Losano JD, Vechiato Kawai GK, Carvalho Celeghini EC (2020). Heat stress effects on bovine sperm cells: A chronological approach to early findings. Int. J. Biometeorol..

[43] Pérez-Crespo M, Pintado B, Gutiérrez-Adán A (2008). Scrotal heat stress effects on sperm viability, sperm DNA integrity, and the offspring sex ratio in mice. Mol. Reprod. Dev..

[44] Boklage CE (1990). Survival probability of human conceptions from fertilization to term. Int. J. Fertil..

[45] Macklon NS, Geraedts JP, Fauser BC (2002). Conception to ongoing pregnancy: The 'black box' of early pregnancy loss. Hum. Reprod..

[46] Wilcox AJ, Weinberg CR, O’Connor JF, Baird DD, Schlatterer JP, Canfield RE, Armstrong EG, Nisula BC (1988). Incidence of early loss of pregnancy. N. Engl. J. Med..

[47] Trivers RL, Willard DE (1973). Natural selection of parental ability to vary the sex ratio of offspring. Science.

[48] Klimada 2.0. https://klimada2.ios.gov.pl/. Accessed 4 Mar 2024 (2024).

[49] Kundzewicz, Z. W. Climate changes, their reasons and effects—Observations and projections. *Landform Anal.* **15**, 39–49. http://yadda.icm.edu.pl/baztech/element/bwmeta1.element.baztech-article-BUJ5-0052-0052. Accessed 4 Mar 2024 (2011).

[50] Błażejczyk, K., Baranowski, J. & Błażejczyk, A. *Wpływ Klimatu na Stan Zdrowia w Polsce: Stan Aktualny Oraz Prognoza do 2100 Roku* (Wydawnictwo Akademickie SEDNO, 2015).

[51] Grzeszczuk-Brendel, H. *Miasto do Mieszkania. Zagadnienia Reformy Mieszkaniowej na Przełomie XIX i XX Wieku i jej Wprowadzenie w Poznaniu w Pierwszej Połowie XX Wieku* (Wydawnictwo Politechniki Poznańskiej, 2012).

[52] Trzeciakowski, L. Społeczeństwo, jego życie codzienne i kultura materialna. In *Dzieje Poznania w Latach 1793–1945* (Topolski, J., Trzeciakowski, L. eds.). 296–320 (Wydawnictwo Naukowe PWN, 1994).

[53] *Die Gemeinden und Gutsbezirke des Preussischen Staates und ihre Bevölkerung. 4, Die Gemeinden und Gutsbezirke der Provinz Posen und ihre Bevölkerung: Nach den Urmaterialien der Allgemeinen Volkszählung vom 1. December 1871* (Verlag des Königlichen Statistischen Bureaus, 1874).

[54] Łuczak, C. *Życie Gospodarczo-Społeczne w Poznaniu 1815–1918* (Wydawnictwo Poznańskie, 1965).

[55] Szulc, W. *Położenie Klasy Robotniczej w Wielkopolsce w Latach 1871–1914* (Wydawnictwo Naukowe UAM, 1970).

[56] Smosarski, W. *Temperatura i Opady w Wielkopolsce* (Ministerstwo Wyznań Religijnych i Oświecenia Publicznego, 1925).

[57] Kolendowicz K, Czarnecki B, Półrolczak B, Taszarek M, Tomczyk AM, Szyga-Pluga K (2019). Homogenization of air temperaturę and its long-term trends in Poznan (Poland) for the period 1848–2016. Theor. Appl. Climatol..

[58] Cunningham JM, Kim CY, Cristensen ER, Tester DJ, Parc Y, Burgart LJ, Halling KC, McDonnel SK, Schaid DJ, WalshVockley C, Kubly V, Nelson H, Michels VV, Thibodeau SN (2001). The frequency of hereditary defective mismatch repair in a prospective series of unselected colorectal carcinomas. Am. J. Hum. Genet..

[59] Bhat RA, Kushtagi P (2006). A re-look at the duration of human pregnancy. Singap. Med. J..

[60] Hastie, T. J. & Tibshirani, R. J. *Generalized Additive Models*. 10.1201/9780203753781 (Boca Raton, 1990).

[61] Wood, S. N. *Generalized Additive Models: An Introduction with R*. 2nd ed. 10.1201/9781315370279 (Boca Raton, 2017).

[62] Wood SN, Pya N, Saefken B (2016). Smoothing parameter and model selection for general smooth models (with discussion). J. Am. Stat. Asoc..

[63] Almon Sh (1965). The distributed lag between capital appropriations and net expenditures. Econometrica.

[64] Brabec M (2018). Semiparametric Model for Short Term Effects of Air Pollution Upon Asthma Symptoms Exacerbations.

[65] Judge GG, Griffiths WE, Hill RC, Lee T-C (1980). The Theory and Practice of Econometrics.

[66] R Core Team. *R: A Language and Environment for Statistical Computing*. https://www.r-project.org/ (R Foundation for Statistical Computing, 2023).

[67] Rocznik Demograficzny 2023 (Główny Urząd Statystyczny, 2023). https://stat.gov.pl/obszary-tematyczne/roczniki-statystyczne/roczniki-statystyczne/rocznik-demograficzny-2023,3,17.html. Accessed 16 June 2024 (2023).

[68] Weng YH, Yang CY, Chiu YW (2015). Neonatal outcomes in relation to sex differences: A national cohort survey in Taiwan. Biol. Sex Differ..

[69] Bohn C, Vogel M, Poulain T, Spielau U, Hilbert C, Kiess W, Körner A (2021). Birth weight increases with birth order despite decreasing maternal pregnancy weight gain. Acta Paediatr..

[70] Chahnazarian A (1988). Determinants of the sex ratio at birth: Review of recent literature. Soc. Biol..

[71] Liczbińska G, Sobkowiak A (2020). Did the sex ratio at birth reflect social and economic inequalities? A pilot study of the Province of Poznań, 1875**–**1913. Poland's Demogr.Past (Przeszłość Demograficzna Polski).

[72] Ruckstuhl KE, Colijn GP, Amiot V, Vinish E (2010). Mother's occupation and sex ratio at birth. BMC Pub. Health.

